# Dynamic Relationship Between Interhemispheric Functional Connectivity and Corticospinal Tract Changing Pattern After Subcortical Stroke

**DOI:** 10.3389/fnagi.2022.870718

**Published:** 2022-05-06

**Authors:** Jingchun Liu, Caihong Wang, Jingliang Cheng, Peifang Miao, Zhen Li

**Affiliations:** ^1^Department of Radiology and Tianjin Key Laboratory of Functional Imaging, Tianjin Medical University General Hospital, Tianjin, China; ^2^Department of MRI, Key Laboratory for Functional Magnetic Resonance Imaging and Molecular Imaging of Henan Province, The First Affiliated Hospital of Zhengzhou University, Zhengzhou, China; ^3^Department of Interventional Radiology, The First Affiliated Hospital of Zhengzhou University, Zhengzhou, China

**Keywords:** cerebral infarction, corticospinal tract, diffusion tensor imaging, functional neuroimaging, motor, neuronal plasticity

## Abstract

**Background and Purpose:**

Increased interhemispheric resting-state functional connectivity (rsFC) between the bilateral primary motor cortex (M1) compensates for corticospinal tract (CST) impairment, which facilitates motor recovery in chronic subcortical stroke. However, there is a lack of data on the evolution patterns and correlations between M1–M1 rsFC and diffusion indices of CSTs with different origins after subcortical stroke and their relations with long-term motor outcomes.

**Methods:**

A total of 44 patients with subcortical stroke underwent longitudinal structural and functional magnetic resonance imaging (MRI) examinations and clinical assessments at four time points. Diffusion tensor imaging was used to extract fractional anisotropy (FA) values of the affected CSTs with different origins. Resting-state functional MRI was used to calculate the M1–M1 rsFC. Longitudinal patterns of functional and anatomic changes in connections were explored using a linear mixed-effects model. Dynamic relationships between M1–M1 rsFC and FA values of the affected specific CSTs and the impact of these variations on the long-term motor outcomes were analyzed in patients with subcortical stroke.

**Results:**

Stroke patients showed a significantly decreased FA in the affected specific CSTs and a gradually increasing M1–M1 rsFC from the acute to the chronic stage. The FA of the affected M1 fiber was negatively correlated with the M1–M1 rsFC from the subacute to the chronic stage, FA of the affected supplementary motor area fiber was negatively correlated with the M1–M1 rsFC in the subacute stage, and FA of the affected M1 fiber in the acute stage was correlated with the long-term motor recovery after subcortical stroke.

**Conclusion:**

Our findings show that the FA of the affected M1 fiber in the acute stage had the most significant correlation with long-term motor recovery and may be used as an imaging biomarker for predicting motor outcomes after stroke. The compensatory role of the M1–M1 rsFC enhancement may start from the subacute stage in stroke patients with CST impairment.

## Introduction

Stroke is the leading cause of significant lifelong motor deficits (Johnson and Westlake, 2021), which adversely affect the clinical outcomes and impair the activities of daily living (Patel et al., 2020). The integrity of motor pathways plays an important role in motor recovery in stroke patients with motor deficits (Guo et al., 2019; Zolkefley et al., 2021). The corticospinal tract (CST) is the principal tract for controlling primary motor activity and has been deemed as the most common locus of motor impairment after subcortical stroke (Schaechter et al., 2009; DeVetten et al., 2010). Diffusion tensor imaging (DTI) is a widely used method for providing information about cellular integrity and pathology (Le Bihan, 2003), analyzing the integrity of white matter fiber tracts (Mori and van Zijl, 2002), and observing the relationship between white matter tracts and infarcts (Kunimatsu et al., 2003; Lee et al., 2005). One of the diffusion indices accessed by DTI is fractional anisotropy (FA), a measure of the white matter tracts' integrity. Considering the CST as a whole tract, a previous longitudinal study showed that the FA values of the affected CST changed dynamically (Yu et al., 2009), and the baseline FA values of the CST can be used to predict the degree of motor recovery (Shaheen et al., 2022). CST fibers arise from the primary motor cortex (M1), premotor cortex (PMC), primary somatosensory area (S1), and supplementary motor area (SMA) (Schieber, 2007; Welniarz et al., 2017). Liu et al. (2020) reconstructed a fine map of the CST fibers with different cortical origins and found that the integrity of M1 and SMA fibers was closely associated with the motor outcomes and structural brain changes. These results suggest that the preserved microstructural integrity of CST affects motor recovery. However, the differences in the evolution patterns of specific CST changes and their correlations with long-term motor recovery in patients with subcortical stroke remain largely unknown. M1 is thought to be the main origin of CST fibers (Seo and Jang, 2013). Therefore, we hypothesize that the FA values of the affected M1 fibers decrease rapidly, impacting the response to motor rehabilitation, and are helpful for understanding the mechanisms of neurological rehabilitation after subcortical stroke.

The resting-state functional connectivity (rsFC) is defined as the statistical dependency among spatially remote neurophysiological events (Friston, 2011), which has been widely used to investigate functional alterations and motor recovery after stroke (Yin et al., 2012). Some cross-sectional studies have suggested a beneficial effect of the restoration or enhancement of the rsFC on the motor function in patients with subcortical stroke (Peng et al., 2019; Chen et al., 2020). The interhemispheric rsFC of M1 has been shown to exhibit different evolution patterns across patients with subcortical stroke. For example, enhanced rsFC between the bilateral M1 areas appears in the first week post-stroke in some patients (Golestani et al., 2013) and starts at a later time (1–12 weeks post-stroke) in some patients (Xu et al., 2014), while it does not show any increase within 1 year after stroke in some patients (Xu et al., 2014). However, the inflection points of M1–M1 rsFC dynamic changes in patients with subcortical stroke remain largely unknown. The first several weeks post-stroke are critical for motor rehabilitation (Verheyden et al., 2008). Therefore, we hypothesize that interhemispheric rsFC reorganization starts in the acute or subacute stage and is critical for selecting the individual time window of intervention in patients with subcortical stroke.

Both integrity of the affected CST and enhanced interhemispheric rsFC are important factors for motor recovery after stroke (Xia et al., 2021). Another critical element is the relationship between these two factors. Previous studies have shown that a decreased M1–M1 rsFC was negatively correlated with the percentage of CST damage within 4 weeks after subcortical stroke (Carter et al., 2012). Liu and coauthors further found that an enhanced interhemispheric rsFC was negatively correlated with the FA values of CST impairment in chronic patients (>6 months) with subcortical stroke (Liu et al., 2015). These cross-sectional studies indicated that the correlations between the two factors were completely opposite in the subacute and chronic stages post-stroke. However, the dynamic relationship between CST impairment and rsFC reorganization in different stages after subcortical stroke remains largely unknown. Moreover, previous studies failed to determine whether the compensatory relationship between enhanced interhemispheric FC and CST damage starts from the inflection point of M1–M1 rsFC changes in subcortical stroke.

In this study, we aimed (a) to explore the evolution patterns of the diffusion indices of specific CSTs in a longitudinal dataset of 44 patients with subcortical stroke; (b) to identify the trajectories of M1–M1 rsFC changes in these patients; (c) to uncover the dynamic relationships between M1–M1 rsFC and diffusion indices of specific CSTs in different stages after stroke and their relationships with long-term motor recovery; and (d) to identify the period of M1–M1 rsFC changes in which the compensatory relationship between enhanced interhemispheric rsFC and CST damage begins in patients with subcortical stroke.

## Materials and Methods

### Subjects

The experimental protocol was approved by the local medical research ethics committee, and written informed consent was obtained from each participant. The study followed a longitudinal design (four time points: ≤7 days, 1 month, 3 months, and >6 months), and the dataset (44 patients and 10 healthy controls) was used to explore the differences in the evolution patterns of specific CSTs and M1–M1 rsFC changes in patients with subcortical stroke.

The inclusion criteria for patients with stroke were as follows: (a) first-onset acute ischemic stroke; (b) a single lesion in the basal ganglia and neighboring regions; and (c) right-handedness before stroke onset (Oldfield, 1971). The exclusion criteria were as follows: (a) recurrent stroke defined by clinical history and magnetic resonance imaging (MRI) evaluation; (b) any other brain abnormalities on MR images; (c) modified Fazekas scale for white matter hyperintensities greater than 1 (Fazekas et al., 1987); and (d) history of any other neurological and psychiatric disorders. During the collection of the dataset, 58 patients with subcortical stroke were initially recruited for this study. Fourteen patients were excluded due to loss of follow-up after inclusion (*n* = 9), recurrent stroke (*n* = 3), and other brain abnormalities (*n* = 2). Finally, 44 patients with data on the four time points were included in this study. All patients with subcortical ischemic stroke were recruited from the Tianjin Medical University General Hospital (*n* = 7) and the First Affiliated Hospital of Zhengzhou University (*n* = 37).

### MR Data Acquisition

Multimodal MRI data were obtained using two 3.0-Tesla Discovery MR750 MR scanners (General Electric, Milwaukee, WI) from two hospitals. Tight but comfortable foam padding was used to minimize head movement, and earplugs were used to reduce the scanner noise. Diffusion-weighted imaging (DWI), sagittal 3D T1- and T2-weighted images, and T2 fluid-attenuated inversion recovery images were acquired to identify the stroke lesion, recurrent stroke, white matter hyperintensity, and other brain abnormalities. DTI was used to extract the FA values of affected CSTs with different origins. The rs-fMRI was used to calculate the M1–M1 rsFC.

Diffusion tensor imaging data were acquired using a spin-echo single-shot echo-planar imaging (EPI) sequence. Diffusion-sensitized gradients were applied along 64 non-collinear directions with a b-value of 1000 s/mm^2^. In addition, three sets of b = 0 images were obtained. Using an integrated parallel acquisition technique (iPAT) with an acceleration factor of 2 allowed us to obtain images with less distortion from susceptibility artifacts. We collected 50 slices from each participant. The scan parameters were repetition time (TR)/echo time (TE) = 11000/77.6 ms; field of view (FOV) = 256 × 256 mm; matrix = 128 × 128; flip angle (FA) = 90°; and slice thickness = 3 mm without gap. Resting-state fMRI data were obtained using a gradient-echo single-shot EPI sequence with the following imaging parameters: TR/TE = 2000/30 ms; FOV = 240 × 240 mm; matrix = 64 × 64; FA = 90°; slice thickness = 3 mm; gap = 1 mm; 38 interleaved transversal slices; and 180 volumes. During the resting-state fMRI scans, all subjects were instructed to keep their eyes closed, stay as still as possible, think of nothing, and not fall asleep. Sagittal 3D T1WI was acquired by brain volume with the following imaging parameters: TR/TE = 8.2/3.2 ms; inversion time = 450 ms; FA = 11°; FOV = 256 mm × 256 mm; matrix = 256 × 256; slice thickness = 1 mm, no gap; and 188 slices. DWI parameters: TR/TE, matrix, FOV, slices, and slice thickness were 3,000 ms/61 ms, 160 × 160, 240 × 240 mm, 20, and 6 mm, respectively, with b-value = 1,000 s/mm^2^. T2-FLAIR parameters: TR/TE, matrix, slices, and slice thickness were 8,500 ms/158 ms, 256 × 256, 20, and 5 mm, respectively. T2WI was acquired from clinically used sequences.

### Behavioral Assessments

For longitudinal analysis of patients with stroke (four time points: ≤7 days, 1 month, 3 months, and >6 months), global functional deficits were assessed using the National Institutes of Health Stroke Scale (NIHSS), and motor outcomes were evaluated by the Fugl–Meyer Assessment of the whole extremity (WE_FM) at four time points.

### Preprocessing of DTI Data

The DTI data were preprocessed using the FMRIB's Diffusion Toolbox (FSL 5.0; http://www.fmrib.ox.ac.uk/fsl). All diffusion-weighted images were visually inspected by two radiologists for apparent artifacts due to subject motion and instrument malfunction. For each subject, the diffusion-weighted images were registered to the corresponding b = 0 images with an affine transformation to correct for eddy-current distortion and motion displacement. Then, skulls in the images were removed using the brain extract toolbox. The diffusion tensor was reconstructed using the linear least-square fitting algorithm, which was used for calculating the diffusion indices. Then, we coregistered the individual diffusion indices into the Montreal Neurological Institute (MNI) space using a two-step method. First, we coregistered the brain-extracted b = 0 images of each subject with his/her T1-weighted images using an affine method (12 parameters), and then, the T1-weighted images were affinely coregistered into the T1 template of the MNI space. Finally, the diffusion indices were written into the MNI space using the affine parameters generated from the above steps and were resliced to 2 × 2 × 2 mm^3^.

The integrity of each CST fiber was assessed by the FA and the percentage of impairment. Based on the fine map of the CST (Liu et al., 2020), the FA values of each CST fiber were extracted from each patient at four time points. Moreover, we only extracted FA values from the cerebral peduncle, where the CST fibers have a relatively coherent arrangement. FA values can accurately estimate the white matter integrity only in the white matter regions with coherent fiber arrangement. We used the mean FA value of all voxels within each CST to represent the FA of the CST. Acute stroke lesions and the fine map (Liu et al., 2020) of the CST fibers were used to calculate the percentage of the impairment of each CST fiber for each patient in the acute stage. First, acute stroke lesions were acquired based on the DWI data. The individual's DWI data were spatially normalized to the EPI template in the MNI space and resampled into a 1 mm^3^ voxel. Stroke lesions were independently outlined on the normalized DWI using the MRIcron tool (https://www.nitrc.org/projects/mricron) by three radiologists with more than 9 years of experience. The intra-class correlation coefficient for the lesion volume was 0.98, and the result from the most senior radiologist was selected as the final lesion contour. Second, for each axial slice with an overlap between the stroke lesion and a given CST fiber, the impairment percentage of the CST fiber at this slice was defined as the ratio of the area of the overlap region to the area of the CST fiber. Finally, the largest percentage in these slices was defined as the impairment percentage of the CST fiber in this patient.

### Seed Masks

The left and right M1s were separately extracted from the Human Brainnetome Atlas (Fan et al., 2016). Subsequently, we separately extracted the overlapping regions of the left and right M1 with the 50% probability fine map (Liu et al., 2020) of the CST to define the left and right seed masks for the rsFC analysis (left: MNI coordinates: −12, −21, 66; cluster size: 61 voxels; right: MNI coordinates: 12, −21, 66; cluster size: 62 voxels).

### Preprocessing of the Resting-State fMRI Data

The resting-state fMRI data were preprocessed using the Statistical Parametric Mapping software (SPM12, http://www.fil.ion.ucl.ac.uk/spm). The first 10 volumes from each subject were discarded to allow the signal to reach equilibrium and to let the participants adapt to the scanning noise. The remaining 170 volumes were corrected for the acquisition time delay between slices. None of the 44 subjects had a maximum displacement of >2 mm or a maximum rotation of >2.0°. The fMRI dataset was spatially normalized to the MNI EPI template and resampled into 3 × 3 × 3 mm^3^ voxels. Thereafter, several nuisance variables were regressed out from the fMRI data, including the averaged signals of the ventricles, white matter, and the whole brain, and the Friston 24 regressors (including six head motion parameters, six head motion parameters one time point before, and the 12 corresponding squared items) (Friston et al., 1996). Then, a band-pass frequency filter (0.01–0.08 Hz) was applied to reduce the low-frequency drift and high-frequency noise (Greicius et al., 2003). Finally, the filtered BOLD images were spatially smoothed using an isotropic Gaussian kernel of 8 mm full width at half maximum (FWHM).

Using the defined seed masks as the regions of interest (ROI), ROI-based rsFC analysis was performed. For each individual dataset, the Pearson correlation coefficient between the mean time series of the left and right ROIs was computed and converted into the z value using Fisher's r-to-z transformation to improve the normality.

### Evolution of CST and rsFC Changes After Stroke

We used a linear mixed-effects model to investigate the evolution patterns of specific CST and M1–M1 rsFC changes in patients with stroke and healthy controls in the longitudinal dataset (44 patients and 10 healthy controls). The random intercept term accounts for the correlation due to repeated measurements within a single patient (Gibbons et al., 1988). All patients were assumed to have a common slope (fixed effect) where only the intercepts were allowed to vary (random effect). The model parameters were estimated by the restricted maximum likelihood method and considered significant if the *P* values were less than 0.05. In healthy controls, we characterized the trajectories of these specific CSTs and M1–M1 rsFC changes to establish references to identify the stroke-induced changes. In both the patient and control groups, we identified significant longitudinal specific CSTs (four fibers from M1, PMC, S1, and SMA) and M1–M1 rsFC changes by assessing the significance of the slopes (five comparisons for the patient group and five comparisons for the control group). For each specific CST and M1–M1 rsFC change, we investigated the differences in the evolution pattern by comparing the slopes between stroke and control groups (five comparisons between the two groups). In the above-mentioned analyses, we performed the comparisons 15 times. To reduce the possible false-positive findings, we used the Bonferroni method (*P* < 0.05/15 = 3.33 × 10^−3^) to correct for multiple comparisons.

### Correlation Analysis

We assessed the relationships between the FA values of the affected specific CSTs and M1–M1 rsFC at four time points. Then, we examined the correlations between the FA values and impairment percentage of each CST fiber in the acute stage and the WE_FM scores in the chronic stage. For all correlation analyses, we used partial correlations to factor out the age, sex, and FA values of the CST fibers with other origins. In the above-mentioned analyses, we performed the correlations 24 times (16 correlations between the FA of the affected CST and M1–M1 rsFC at four time points, and eight correlations between the integrity of the affected CST and long-term motor outcomes). To reduce the possible false-positive findings, we used the Bonferroni method (*P* < 0.05/24 = 2.08 × 10^−3^) to correct for multiple comparisons.

## Results

### Demographic and Clinical Information

**Figure 1:**
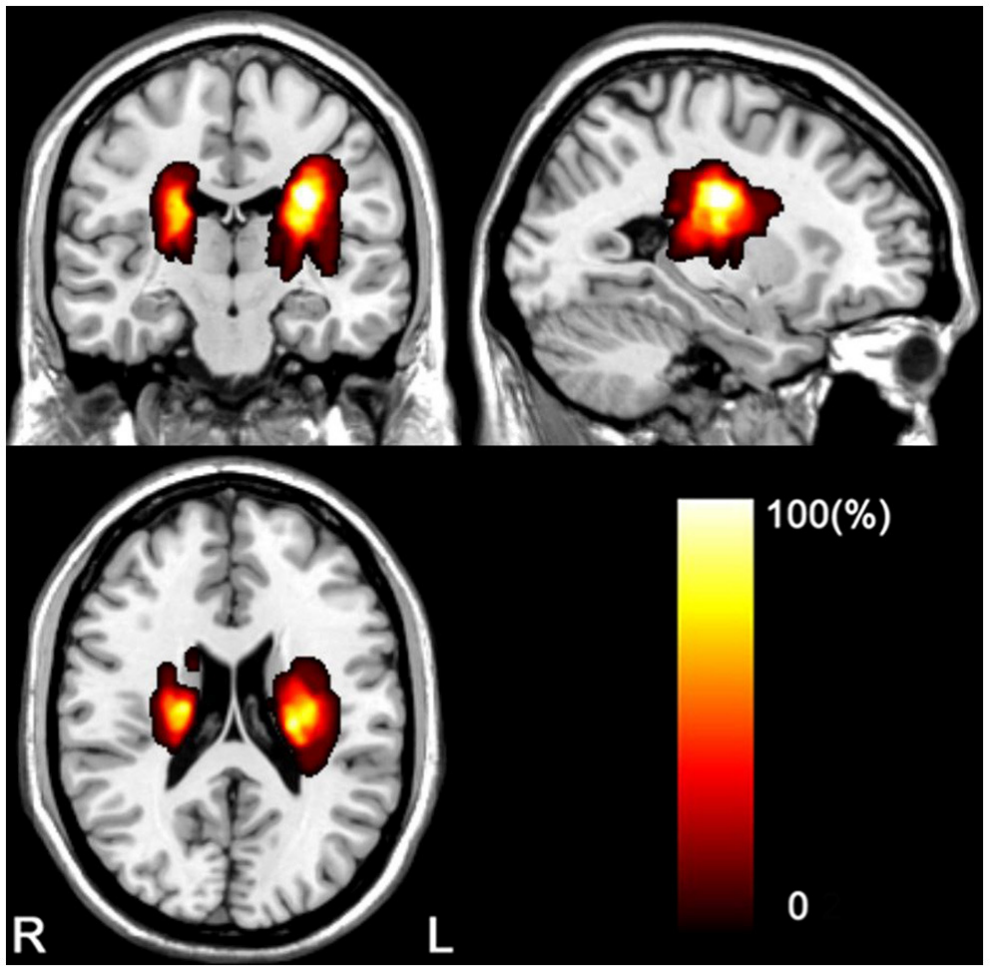
Lesion incidence map of patients with stroke. L, left; R, right.

The clinical and demographic data of the stroke patients and controls are listed in Table 1. The longitudinal dataset (44 patients with subcortical stroke and 10 healthy controls) included rs-fMRI, DTI, and WE_FM data from the acute to chronic stages. The stroke lesions involved the internal capsule and surrounding structures, including the internal capsule, thalamus, basal ganglia, and corona radiata (Figure 1). A total of 24 out of 44 patients had infarct lesions in the right hemisphere and 20 in the left hemisphere. The motor function of the patients was partially or completely recovered with an FMA > 52/100 for the whole extremities.

**Table 1:** Demographic and clinical information of the participants.

**Variables**	**Patients with subcortical stroke** **(*n =* 44)**	**Healthy controls** **(*n =* 10)**
Age, y	53.8 ± 8.8 (30–72)	55.9 ± 5.2 (48–66)
Sex (M/F)	33/11	3/7
**Time points**		
1, d	6 (2–7)	0 (0–0)
2, d	35 (31–40.8)	44.5 (33.3–59)
3, mo	3.3 (3.1–3.5)	4.9 (4.3–6.6)
4, mo	6.5 (6.1–7.2)	8.5 (7.1–11.6)
**Lesion location**		
Left hemisphere	20 (45.5%)	
Right hemisphere	24 (54.5%)	
**NIHSS**		
1	2 (2–3)	
2	1 (1–2)	
3	1 (0–2)	
4	0 (0–1)	
**WE_FM**		
1	89.4 ± 19.3 (15–100)	
2	93.5 ± 16.7 (20–100)	
3	96.1 ± 11.9 (39–100)	
4	96.2 ± 9.9 (52–100)	

*Data are presented as means ± SD (range) for continuous data, medians (Q1-Q3) for time points and NIHSS, and n (%) for categorical data. NIHSS, National Institutes of Health Stroke Scale; WE_FM, Fugl–Meyer Assessment of the whole extremity*.

### Evolution of CST Changes After Stroke

**Figure 2:**
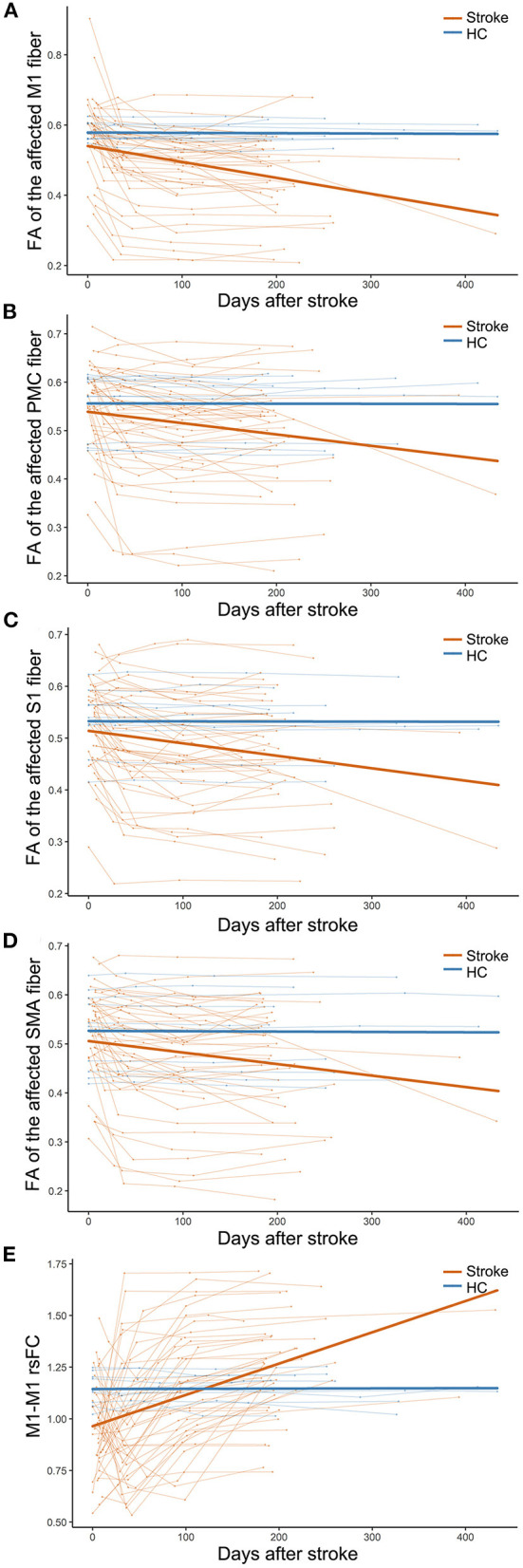
Trajectories of CST and M1–M1 rsFC changes in patients with subcortical stroke. **(A–D)** Show the longitudinal evolutionary trajectories of the CSTs with different origins. **(E)** Shows the longitudinal evolutionary trajectories of M1–M1 rsFC. The thin red (stroke) and blue (HC) lines represent the individual changes over time and the thick red and blue lines indicate the estimated average changes in the two groups. CST, corticospinal tract; FA, fractional anisotropy; HC, healthy controls; M1, primary motor cortex; PMC, premotor cortex; rsFC, resting-state functional connectivity; S1, primary sensory area; and SMA, supplementary motor area.

In 44 subcortical stroke patients with longitudinal DTI data, we observed longitudinal changes in the CSTs from different origins. The trajectories of the specific CST changes are shown in Figure 2. The statistical significance of the longitudinal specific CST changes in the stroke and control groups and the slope differences in the specific CST changes between the two groups are provided in Table 2. For the M1 fiber (Figure 2A), the stroke group showed a significant longitudinal change (decline over time; *P* = 2.83 × 10^−7^, Bonferroni's correction) and had a steeper slope (*P* = 4.00 × 10^−17^, Bonferroni's correction) than the control group. For the PMC fiber (Figure 2B), the stroke group demonstrated a steeper slope (*P* = 7.08 × 10^−8^, Bonferroni's correction), but the longitudinal change (decline over time; *P* = 5.76 × 10^−3^ > 0.05/15 = 3.33 × 10^−3^, Bonferroni's correction) did not differ between the groups. For the S1 fiber (Figure 2C), the stroke group showed a significant longitudinal change (decline over time; *P* = 3.32 × 10^−4^, Bonferroni's correction) and had a steeper slope (*P* = 2.43 × 10^−11^, Bonferroni's correction) than the control group. For the SMA fiber (Figure 2D), the stroke group showed a longitudinal change (decline over time; *P* = 3.27 × 10^−3^, Bonferroni's correction) and had a steeper slope (*P* = 8.33 × 10^−9^, Bonferroni's correction) than the control group.

**Table 2:** Longitudinal evolution patterns of CST and M1–M1 rsFC changes after subcortical stroke.

**Variables**	**Stroke**	**HC**	**Stroke vs. HC**
**FA value of CST**			
M1 fiber	**4.00 × 10** ^−17*^	0.90	**2.83 × 10** ^−7*^
PMC fiber	**7.08 × 10** ^−8*^	0.97	5.76 × 10^−3^
S1fiber	**2.43 × 10** ^−11*^	0.96	**3.32 × 10** ^−4*^
SMA fiber	**8.33 × 10** ^−9*^	0.91	**3.27 × 10** ^−3*^
**M1–M1 rsFC**	**1.11 × 10** ^−16*^	0.97	**1.55 × 10** ^−6*^

Data are presented as the uncorrected or corrected P values. Bold values and * indicates that the result remained valid after Bonferroni's correction (*P < 0.05/15 = 3.33 × 10^−3^). CST, corticospinal tract; FA, fractional anisotropy; HC, healthy controls; M1, primary motor cortex; PMC, premotor cortex; rsFC, resting-state functional connectivity; S1, primary sensory area; SMA, supplementary motor area; vs, versus.

### Evolution of M1–M1 rsFC Changes After Stroke

In 44 subcortical stroke patients with longitudinal resting-state fMRI data, we observed longitudinal M1–M1 rsFC changes. The trajectories of the M1–M1 rsFC changes are shown in Figure 2. The statistical significance of the longitudinal M1–M1 rsFC changes in the stroke and control groups and the slope differences in the M1–M1 rsFC changes between the two groups are provided in Table 2. With respect to M1–M1 rsFC (Figure 2E), the stroke group exhibited a longitudinal change (an increase over time; *P* = 1.55 × 10^−6^, Bonferroni's correction) and had a steeper slope (*P* = 1.11 × 10^−16^, Bonferroni's correction) than the control group.

### Correlation Analyses

**Figure 3:**
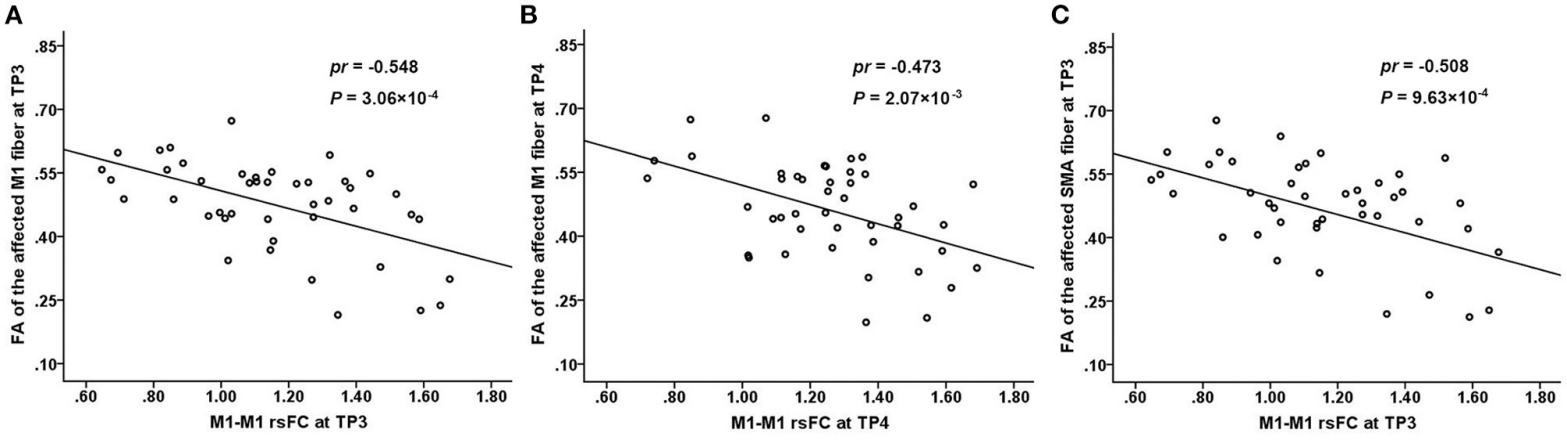
Correlations between the connectivity measures. There is a significantly negative correlation between the FA values of the affected M1 fiber and M1–M1 rsFC at TP3 (3 months post-stroke) **(A)** and TP4 (>6 months post-stroke) **(B)** after subcortical stroke. There is a significantly negative correlation between the FA values of the affected SMA fiber and M1–M1 rsFC at TP3 (3 months post-stroke) **(C)** after subcortical stroke. FA, fractional anisotropy; M1, primary motor cortex; rsFC, resting-state functional connectivity; SMA, supplementary motor area; TP, time point.

The FA values of the M1 fiber were significantly negatively correlated with the M1–M1 rsFC in the subacute stage (3 months post-stroke) (partial correlation coefficient [*pr*] = −0.548, *P* = 3.06 × 10^−4^) (Figure 3A) and chronic stage (> 6 months post-stroke) (*pr* = −0.473, *P* = 2.07 × 10^−3^) (Figure 3B) after subcortical stroke. Similarly, a negative correlation was observed between the FA values of the SMA fiber and M1–M1 rsFC in the subacute stage (3 months post-stroke) (*pr* = −0.508, *P* = 9.63 × 10^−4^) (Figure 3C) after subcortical stroke. However, none of the FA values of the affected PMC and S1 fibers were correlated with the M1–M1 rsFC at the four time points after subcortical stroke (*P* > 0.05/24 = 2.08 × 10^−3^, Bonferroni's correction).

**Figure 4:**
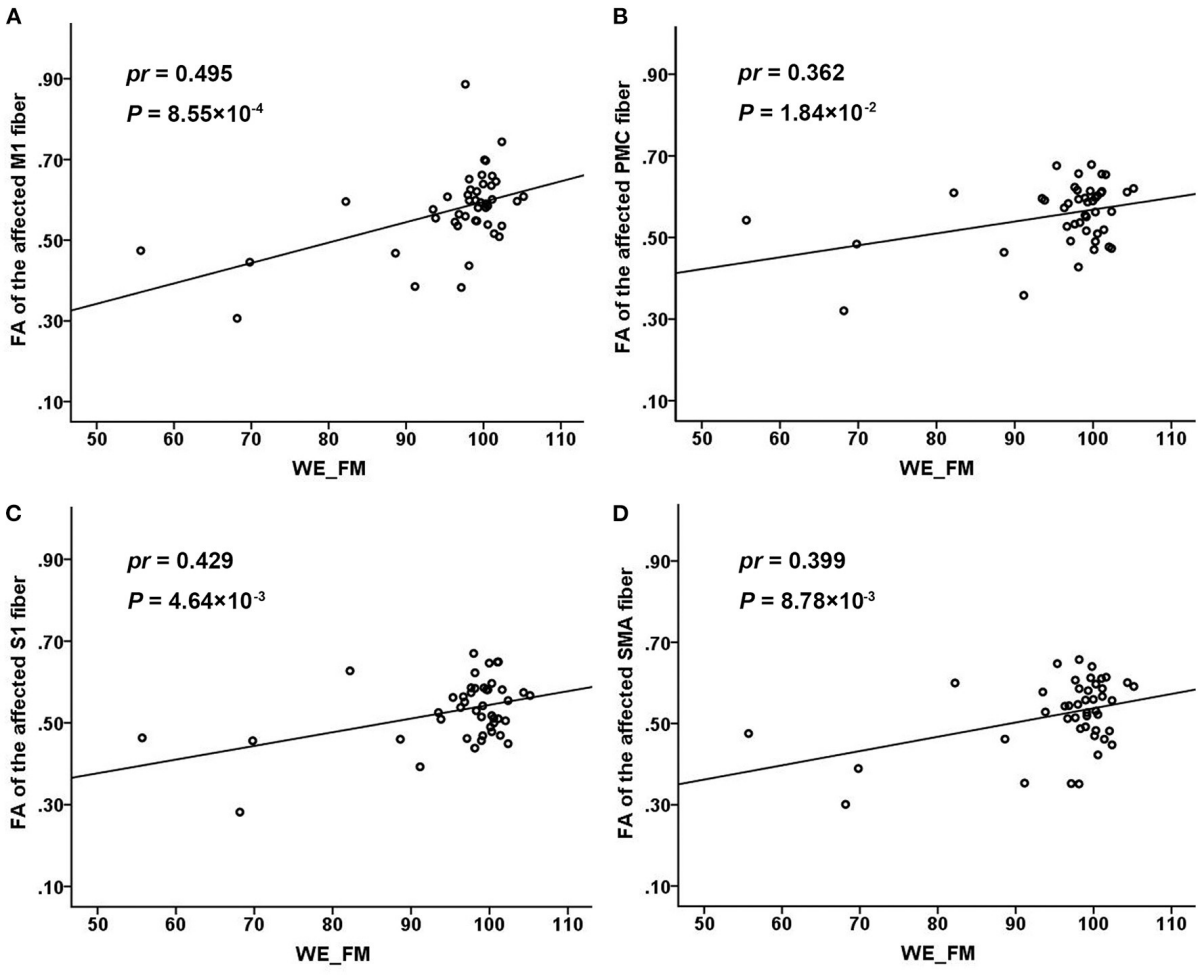
Associations of the FA values of different CST fibers with chronic WE_FM scores in subcortical stroke. The FA values of the affected M1 **(A)**, PMC **(B)**, S1 **(C)**, and SMA **(D)** fibers in the acute stage were positively correlated with the WE_FM scores in the chronic stage. CST, corticospinal tract; FA, fractional anisotropy; M1, primary motor cortex; PMC, premotor cortex; S1, primary sensory area; SMA, supplementary motor area; WE_FM, Fugl–Meyer Assessment of the whole extremity.

In the 44 patients with subcortical stroke, the FA values of the affected M1 fiber in the acute stage were positively correlated with the WE_FM scores in the chronic stage (*pr* = 0.495, *P* = 8.55 × 10^−4^, Bonferroni's correction; Figure 4A). However, none of the FA values of the affected PMC (*pr* = 0.362, *P* = 1.84 × 10^−2^, Bonferroni's correction; Figure 4B), S1 (*pr* = 0.429, *P* = 4.64 × 10^−3^, Bonferroni's correction; Figure 4C), and SMA (*pr* = 0.399, *P* = 8.78 × 10^−3^, Bonferroni's correction; Figure 4D) fibers was correlated with the WE_FM scores in the chronic stage (*P* > 0.05/24 = 2.08 × 10^−3^, Bonferroni's correction). None of the early impairments of the affected M1, PMC, S1, and SMA fibers were correlated with the WE_FM scores in the chronic stage (*P* > 0.05/24 = 2.08 × 10^−3^, Bonferroni's correction).

## Discussion

In this study, we found significantly reduced FA values of the affected specific CST fibers and increased M1–M1 rsFC. The enhanced M1–M1 rsFC values were negatively correlated with the FA values of the affected M1 fiber from the subacute to the chronic stage and negatively with the FA values of the affected SMA fiber in the subacute stage. These findings suggest that early interhemispheric functional reorganization may play a beneficial role in motor rehabilitation. We also found that the FA values of the affected M1 fiber in the acute stage were significantly correlated with the motor recovery in the chronic stage, indicating that the affected M1 fiber was critical to predicting the long-term motor outcomes after subcortical stroke.

Regarding CST as a whole tract, a previous study found that the ratios of the FA values decreased continuously during the first 3 months and then stabilized in patients with subcortical stroke (Yu et al., 2009). However, the CST fibers arise from M1, PMC, SMA, and S1 areas (Schieber, 2007; Welniarz et al., 2017). Considering the functional differences among these CST fibers with different origins, the differential involvement of the CST branches may result in distinct functional deficits. We further analyzed the dynamic evolution of the diffusion indicators of the affected specific CST fibers. According to the average changes in the affected specific CSTs, we found that the FA values of M1, S1, and SMA fibers decreased significantly and rapidly after subcortical stroke, which might reflect the pathological process of Wallerian degeneration (Yu et al., 2009). For each individual change, the FA values of the CST fibers decreased rapidly within 1 month after stroke, then slowly reduced from 1 month to 3 months, followed by stabilization after 3 months in patients with subcortical stroke. Furthermore, the FA values of the affected M1 fiber decreased more rapidly than those of other fibers. The M1 has been considered the main origin of the CST, and the affected M1 fiber seems to be more vulnerable to direct damage by stroke lesions after subcortical stroke than the other CST fibers.

In subcortical stroke patients, the evolution patterns of M1–M1 rsFC changes were complex in different individuals. Previous studies have shown that some patients exhibit a decrease in the rsFC of bilateral M1 within 24 h after stroke (Golestani et al., 2013), while it may be at a later time (1–2 weeks) in other patients (Xu et al., 2014). Consistent with these findings, we also found that most of the patients with subcortical stroke exhibited a decrease in the interhemispheric rsFC of M1 at an initial stage. According to the existing literature (Feeney and Baron, 1986; Andrews, 1991), the initial decrease in the M1–M1 rsFC may be caused by an imbalance in the interhemispheric activity changes, which has been found in the first week after stroke in rats (Jablonka et al., 2010). However, few patients showed an increase in the rsFC at the first time point. This might be explained by the later first time point (7 days post-stroke) of MRI acquisition in a few patients. In order to observe the changes rapidly, it is necessary to add time points in the acute stage in a future study. In the final stable stage (nearly 1 year post-stroke), some studies have suggested that the interhemispheric rsFC of M1 returns to a normal level in some patients (Wang et al., 2010; Urbin et al., 2014), reaches a greater than normal level in some others, and remains at a lower level in some patients (Xu et al., 2014). Similarly, we also found different changes in the M1–M1 rsFC in the chronic stage (>6 months) after subcortical stroke. The restoration of the M1–M1 rsFC may be related to the disappearance of the temporary transhemispheric diaschisis (Andrews, 1991), sprouting of axons to establish new connections and novel projection patterns (Carmichael, 2006, 2008), and/or functional reorganization within the motor network (Wang et al., 2010).

For CST as a whole tract, a previous study found that the percentage of CST damage was negatively correlated with the interhemispheric rsFC reduction in subacute stroke patients (<4 weeks post-stroke) with subcortical lesions (Carter et al., 2012). However, we did not find any significant correlations between the FA values of CST impairment and M1–M1 rsFC in early subacute patients with subcortical stroke. This discrepancy may be explained by the difference in the time points of MRI acquisition between the two studies. Our patients were scanned at <7 days, 1 month, 3 months, and >6 months post-stroke. Liu et al. (2015) found that the M1–M1 rsFC increase was negatively correlated with the CST damage in patients with chronic subcortical stroke (>6 mouths post-stroke). The result of this cross-sectional study is consistent with our finding of a negative correction between these two measures in chronic patients with subcortical stroke. This finding indicates that the CST impairment from a focal stroke lesion could be compensated by the enhanced interhemispheric rsFC of M1, which may reflect a form of reactive functional reorganization. We further found that an increased M1–M1 rsFC is negatively correlated with impairment of the affected M1 and SMA fibers starting from the subacute stage (3 months post-stroke) after stroke, indicating that the rehabilitation of enhanced interhemispheric rsFC possibly occurs in the subacute stage after subcortical stroke. This finding suggests that the rehabilitation of M1–M1 rsFC enhancement may be beneficial for the motor recovery of stroke patients with CST injury, which has important clinical implications, as this kind of rehabilitation may be more efficacious if patients can strengthen rehabilitation training or physical stimulation (e.g., transcranial magnetic stimulation, transcranial direct current stimulation) in these regions in the subacute stage. This will also be our goal in our future studies.

The microstructure integrity of the CSTs is correlated with motor recovery in patients with subcortical stroke (Liu and Wang, 2022). The M1 is the main site of origin of the CST, and the M1 fiber seems to be more important for motor function than the other CST fibers (Schieber, 2007; Welniarz et al., 2017; Liu et al., 2020). Liu et al. (2020) found that the early impairments of M1 and SMA fibers were significantly associated with motor deficits in chronic stroke patients. In our study, we also provided sufficient evidence on the importance of the M1 fibers for motor function. For example, the FA values of the affected M1 fiber were correlated with the long-term motor outcomes. However, we did not find any significant correlations for CST fibers originating from the SMA, S1, and PMC, indicating that these fibers make a limited contribution to motor deficits assessed by clinical motor scales. In general, these findings indicate that the M1 fiber is a reliable predictive indicator of the long-term motor outcome in patients with subcortical stroke. In addition, we also assessed the integrity of the M1 fibers in the acute stage, which may help to predict the clinical benefits of different rehabilitative strategies. For example, if the M1 fibers are completely interrupted in patients with subcortical stroke, a little clinical benefit can be obtained from physical stimulation targeting the M1 areas. In this situation, alternative rehabilitative strategies which can enhance an alternative motor pathway (Ruber et al., 2012; Schulz et al., 2017) or establish new motor fibers (Carmichael et al., 2017) may be preferable.

In this study, the evolution patterns of affected CST fibers with different cortical origins and M1–M1 rsFC and their dynamic relationships were studied in patients with subcortical stroke. The enhanced interhemispheric rsFC of M1 may reflect a compensatory mechanism for motor deficits caused by the impairment of the affected M1 and SMA fibers from the subacute to chronic stages. FA values of the affected M1 fiber in the acute stage were significantly correlated with the long-term motor recovery, which may be used to screen imaging biomarkers for predicting motor outcomes. These findings may be helpful in the design of individualized rehabilitative plans.

## Data Availability Statement

The raw data supporting the conclusions of this article will be made available by the authors, without undue reservation.

## Ethics Statement

The studies involving human participants were reviewed and approved by Tianjin Medical University General Hospital medical research Ethics Committee. The patients/participants provided their written informed consent to participate in this study.

## Author Contributions

JL and CW contributed to the conception and design of the study. JL and JC performed the experiments and analyzed the data. ZL and PM were involved in the clinical assessment. JL wrote the first draft. All authors contributed to the manuscript revision and approved the submitted version.

## Funding

This study was supported by the Natural Science Foundation of China (81871327 and 82030053), the Tianjin Key Technology R&D Program (17ZXMFSY00090), the Tianjin Key Medical Discipline (Specialty) Construction Project, and the Young Talents Promotion Program of Henan Province (2021HYTP012).

## Conflict of Interest

The authors declare that the research was conducted in the absence of any commercial or financial relationships that could be construed as a potential conflict of interest.

## Publisher's Note

All claims expressed in this article are solely those of the authors and do not necessarily represent those of their affiliated organizations, or those of the publisher, the editors and the reviewers. Any product that may be evaluated in this article, or claim that may be made by its manufacturer, is not guaranteed or endorsed by the publisher.

## Abbreviations

CST: corticospinal tract
DTI: diffusion tensor imaging
DWI: diffusion-weighted imaging
EPI: echo-planar imaging
FA: fractional anisotropy
FOV: field of view
M1: primary motor cortex
MNI: Montreal Neurological Institute
MRI: magnetic resonance imaging
NIHSS: National Institutes of Health Stroke Scale
PMC: premotor cortex
ROI: region of interest
rsFC: resting-state functional connectivity
S1: primary somatosensory area
SMA: supplementary motor area
TE: echo time
TR: repetition time
WE_FM: Fugl–Meyer Assessment of the whole extremity.
